# "The Silent Struggle: Neurocognitive Impairment and Epilepsy in Children After Severe Malaria in Endemic Regions"

**DOI:** 10.1002/alz70857_104645

**Published:** 2025-12-25

**Authors:** Leonard Ngarka, Constance Ayuk Agbor, Evelyne Mah, Leonard Nfor Njamnshi, Alfred K Njamnshi

**Affiliations:** ^1^ Yaounde Central Hospital, Yaounde, Centre, Cameroon; ^2^ Yaounde Central Hospital, Yaounde, Centre Region, Cameroon; ^3^ Faculty of Medicine and Biomedical Sciences, Yaounde, Centre Region, Cameroon; ^4^ State‐owned Public Teaching Hospital, Yaounde, Centre Region, Cameroon

## Abstract

**Background:**

Severe malaria remains a significant public health burden, particularly in sub‐Saharan Africa. Although effective treatments have improved survival rates, sequelae such as neurocognitive impairment (NCI) and epilepsy can have chronic effects on academic performance, social interactions, and quality of life.

**Method:**

To determine the prevalence and risk factors of cognitive impairment and epilepsy, as well as the EEG patterns in children who have recovered from severe malaria in various hospitals in Yaoundé, Cameroon. The Purdue Pegboard Test, California Verbal Learning Task, ColorTrails1 and ColorTrails2 were used for cognitive evaluation.

**Result:**

Fifty children (27 males, 23 females) were included in the study. The prevalence of NCI was 38%, and the prevalence of epilepsy was 20%. Crude incidence rates per 1,000 person‐years were 226 for NCI, 140 for epilepsy, and 154 for EEG epileptic activity. EEG abnormalities were found in 59% of those tested. The risk of epilepsy associated with EEG epileptic activity was 34.4%. Significant associations were observed between seizure frequency and epilepsy (*p* = 0.004, 0.013) and between disease duration and EEG epileptic activity (*p* = 0.043). A borderline association was found between coma and NCI (*p* = 0.054).

**Conclusion:**

Our results suggest that children exposed to severe malaria are predisposed to neurocognitive impairment and epilepsy. Furthermore, high seizure frequency and disease duration are predictors of epilepsy, while coma serves as a mild predictor of neurocognitive impairment.

**Keywords**: Severe malaria, neurocognitive impairment, epilepsy

